# Effect of Diet on the Midgut Microbial Composition and Host Immunity of the Fall Armyworm, *Spodoptera frugiperda*

**DOI:** 10.3390/biology11111602

**Published:** 2022-11-01

**Authors:** Xiaoxia Xu, Surajit De Mandal, Hongxin Wu, Shaojie Zhu, Jinrong Kong, Sisi Lin, Fengliang Jin

**Affiliations:** Laboratory of Bio-Pesticide Creation and Application of Guangdong Province, College of Plant Protection, South China Agricultural University, Guangzhou 510642, China

**Keywords:** *Spodoptera frugiperda*, gut microbiota, high-throughput 16S rRNA sequencing, PICRUSt

## Abstract

**Simple Summary:**

Gut microbiota plays an important role in the colonization of insects in a new environment. The present study explored the effect of different diets on the midgut microbial composition and host immunity in *Spodoptera frugiperda*. A significant difference was observed in the gut microbiota of *S. frugiperda* feeding on corn leaf (field diet) compared to those on a starch-rich artificial diet (lab diet). Furthermore, an antibiotic-mediated perturbation of the midgut microbiota significantly impacts the expression profile of the important immune genes. Overall, this study reveals that diet composition affects the insect gut microbiome and immune gene expression, ultimately playing an important role in the pest defense system.

**Abstract:**

The fall armyworm (*Spodoptera frugiperda*, J.E. Smith) is one of the most important agricultural pests in the world and causes serious damage to many significant crops. Insect gut microbiota plays a vital role in host immunity, digestion, and development, helping the higher organism colonize in a new environment. However, the effects of different diets on midgut microbial composition and host immunity in *S. frugiperda* remain unclear. So far, no reports have compared the gut microbiota of fall armyworm reared using an artificial diet compared to corn leaf in Guangzhou, China. High-throughput 16S rRNA sequencing technology was applied to gain insight into the composition of the gut microbiota of *S. frugiperda* feeding on corn leaf (field diet) and on a starch-rich artificial diet (lab diet). The fall armyworm gut microbiota was dominated by the bacterial phyla Firmicutes and Proteobacteria. Despite the difference in diet, the core bacterial community was represented by the genus *Enterococcus.* However, the bacterial community is dominated by a few phylotypes, namely operational taxonomical units 1 (OTU1) (*Enterococcus casseliflavus*), OTU3 (*Enterobacteriaceae*), OTU2 (*Weissella*), and OTU4 (*Clostridium*), accounting for 97.43% of the total OTUs in the complete dataset. A significant difference was identified in the bacterial communities between the “lab diet” and the “field diet” groups. OTU1 and OTU2 were significantly higher in the “field diet” group, whereas OTU3 and OTU4 were higher in the “lab diet” group. A phylogenetic investigation of the communities by reconstruction of unobserved states (PICRUSt) predicted functional analysis indicates the presence of several genes associated with plant biomass degradation. Importantly, antibiotic-mediated perturbation of the midgut microbial community significantly impacts the expression profile of the important immune genes of the host. Furthermore, the oral reintroduction of gut bacterial isolates (*E. mundtii* and *E. gallinarum*) significantly enhances host resistance to AcMNPV infection. Taken together, our results indicate that diet composition is an important driver in shaping insect gut microbiome and immune gene expression, ultimately playing an important role in the pest defense system.

## 1. Introduction

Insects represent the most successful taxon of eukaryotic life and exhibit a diverse set of niches on earth. Insects are colonized by a wide group of microorganisms, and the cuticle and gut are the major habitats of bacteria [1]. While the cuticle is the first barrier against commensals or pathogens [2], insect gut microorganisms play a significant role in immunity, digestion, and the host development process, thereby helping the higher organism to colonize a new environment [3,4,5]. The larval stage of the pest can consume large amounts of plant materials. Therefore, they often face various challenges, such as nutritionally recalcitrant food sources, toxins, environmental extremes, and threats from parasites and pathogens [6,7]. It was also found that the larval growth rate can be influenced by the gut microbial composition or the combined effect of the microbiota and the host plant species. Several studies have shown that gut bacteria significantly affect the physiological functions of the Lepidopteran [8,9,10,11]. However, the functional role of the Lepidopteran gut microbiota is challenged due to the presence of no or few resident bacteria in the caterpillars as compared to other insect orders [12]. Several factors, such as the presence of an unusually alkaline gut, the low retention time of food, and the reshaping of body structures through holometabolous metamorphosis, may prevent Lepidopterans from forming robust “core” microbiomes [12].

The fall armyworm *Spodoptera frugiperda* (J.E. Smith) (Lepidoptera: Noctuidae) is considered one of the most significant agricultural pests in the world. It is native to the tropical and subtropical regions of the Americas and damages more than 350 species of plants, including many important crops, such as barley, buckwheat, corn, sorghum, rice, wheat, etc. [13,14]. The first reports of the *S. frugiperda* invasion in Africa were reported in January 2016, and since then, it has spread to Myanmar, Bangladesh, Thailand, Yemen, Sri Lanka, and China [15,16,17,18,19]. By July 2019, this notorious pest had spread to 19 provinces of China [20]. Information on the interaction between *S. frugiperda* and the host as well as the factors associated with the rapid spread of *S. frugiperda* across different geographical regions is limited. A possible factor that helped them rapidly spread and adapt to new environments is their polyphagous nature, which helps them to attack a wide array of hosts, produce many eggs, and migrate long distances [21]. Several techniques have been applied to control different pests, including the sterile insect technique (SIT), chemical insecticides, and biological pest control [22,23]. The SIT is used to produce sterile insects by disrupting their natural reproductive processes, allowing them to mate with native insects, which reduces native insect populations.

However, the effectiveness of most synthetic insecticides against *S. frugiperda* is limited because larvae can hide inside the plant whorl and have a developed resistance against commonly used chemical insecticides such as pyrethroids, organophosphates, and carbamates. Moreover, *S. frugiperda* has also developed a field-evolved resistance against Bt maize [24,25]. Consequently, an alternate, cost-effective, environmentally friendly strategy is urgently needed to control this destructive pest. Increasing evidence on the gut microbiome of different lepidopterans and altering insects’ microbiomes has been considered an effective way to control pests against plant diseases [26]. The coevolution of insects and their gut microbes helps the host to digest plant secondary metabolites, polymers, and produce by-products and biofuels [27]. It has been reported that the gut microbiota of the garden pest *Brithys crini* enables the pest to withstand the toxic compounds (alkaloids) present in the plant *Pancratium maritimum* [28]. Similarly, the gut microbe of the primary coffee bean pest *Hypothenemus hampei* is associated with the degradation of caffeine in coffee beans, enabling the pest to overcome the plant’s toxicity. Ceja-Navarro et al. (2015) proposed that targeting the gut microbiota could be a way to combat these pests [29]. However, the roles of gut microbiota in facilitating plant–insect interactions in most lepidopteran pests are poorly characterized.

Targeting the resident’s microbiota has great potential to improve control methods, and therefore it is very important to study the composition and functional role of the *S. frugiperda* gut microbiota [30]. Despite the economic importance of fall armyworms, little is known about the gut microbial communities and their functional attributes, which may be a critical factor in their rapid spread and adaptation to new environments [31,32]. Analysis of bacterial isolates from field-collected *S. frugiperda* larvae indicates that these isolates could regulate plants’ defensive proteins [33]. Almeida et al. (2017) revealed the presence of different insecticide-degrading bacteria in the gut of the fifth instars of *S. frugiperda* strains resistant to different pesticides [34]. The metatranscriptomic-based study revealed the presence of novel genes and active components in the gut microbiota of *S. frugiperda* that played an important functional role and identified potential biocontrol agents [35]. Moreover, the analysis of the bacterial community and the identification of the important bacterial genus of the *S. frugiperda* collected from the USA, Brazil, Kenya, Argentina, and Nigeria have been documented [33,34,35,36,37]. The present study characterizes the gut microbiome associated with the Lepidopteran pest *S. frugiperda* in China. In order to highlight the role of gut microbiota in the damage of maize, we studied two different populations of *S. frugiperda* feeding on corn leaf (field diet) and on starch-rich artificial foods (lab diet). A comparison of the gut bacterial community and the host immunity of *S. frugiperda* feeding on the different field and lab diets was conducted. Specifically, our objectives were to characterize and compare the midgut microbial community of the field diet and lab diet group and predict their functional roles. This study also investigates how the gut microbiome affects the host immune gene expression. Therefore, evaluating pest-associated microbiomes and their role in immune gene expression under differential diets will be an important foundation for exploring the insect–microbiome interactions that may be exploited to improve pest control strategies.

## 2. Materials and Methods

### 2.1. Sample Collections

The field population of *S. frugiperda* larvae feeding on corn leaf were collected from infested corn fields in South China Agricultural University (SCAU) farm, Guangzhou, China in July 2019. The collected **S. frugiperda* larvae* were divided into two parts. Some were fed corn leaves (field diet), and others were fed an artificial diet rich in starch (lab diet) at 25 ± 2 °C, 70–80% relative humidity, and a 16 h light/8 h dark photoperiod without exposure to insecticides over ten generations. The composition of the lab diet was soybean powder (100 g), wheat bran (80 g), yeast powder (26 g), casein (8 g), ascorbic acid (8 g), agar (26 g), distilled water (1000 mL), choline chloride (1 g), sorbic acid (2 g), and 0.2 g inositol (0.2 g). Pupae were collected and transferred to another bottle, where emerging adults were fed a 10% honey solution to mate and lay eggs. Laboratory-reared larvae and field-collected larvae were simultaneously treated with 75% ethanol and washed with autoclaved ddH_2_O at 10 am on the second day of the third instar stage. Larvae were paralyzed using cold shock and dissected in phosphate-buffered saline (PBS) using sterilized dissection tools. Specifically, a total of ten samples (each comprising five laboratory-reared and field-collected larvae), each consisting of the guts from 30 third instar individuals, were used in this study. Following dissection, the midgut samples were collected in sterile conditions for the subsequent experiments.

### 2.2. Midgut DNA Extraction

The metagenomic DNA from the midgut sample was extracted with the QIAamp DNA Stool Mini Kit (QIAGEN, Hilden, Germany) according to the manufacturer’s protocol. The quantity and purity of DNA were analyzed using a Nanodrop spectrophotometer (Thermo Fisher Scientific, Wilmington, DC, USA). Quantified samples were immediately used for the following experiments or stored at −80 °C. DNA extracted from gut samples was used as a template for the amplification of the V4 hypervariable region of the bacterial 16S rRNA using the 515f/806r primer set (515f, GTGCCAGCMGCCGCGGTAA; 806r, GGACTACHVGGGTWTCTAAT). Sequencing was conducted on an Illumina HiSeq platform (BGI, Shenzhen, China). 

### 2.3. Bioinformatics Analysis

Sequence reads were checked for quality using FastQC [38], and pre-processed to remove reads having low quality, low complexity, containing N, etc. [30,39,40]. Paired-end sequences were merged using the FLASH software (v1.2.11). Operational taxonomical units (OTUs) were generated using USEARCH (v7.0.1090) based on the 97% sequence similarities [41]. UCHIME (v4.2.40) was used to remove the chimera generated by PCR amplification from the OTU-representative sequence [42]. Alpha-diversity indices were calculated using the rarefied OTUs table in QIIME. A normalized OTUs table was prepared by a closed reference-based approach using Greengenes ver. 13.5 databases for the functional prediction. Finally, phylogenetic investigation of communities by reconstruction of unobserved states (PICRUSt) was used to predict the imputed function of the gut bacterial communities based on 16S rRNA marker gene sequences [43].

### 2.4. Antibiotic Treatment and Removal of the Gut Bacterial Communities

Antibiotic treatment was performed by oral feeding to the *S. frugiperda* larvae grown on corn leaves (field diet) and artificial diet (lab diet), and the four experimental conditions involved field diet with antibiotics (YK), field diet without antibiotics (Y), lab diet with antibiotics (SK), lab diet without antibiotics (S). The final antibiotic solution (80 μg/mL) was prepared by mixing seven antibiotics such as neomycin sulfate (50 mg/mL), chloramphenicol (50 mg/mL), gentamycin sulfate (50 mg/mL), streptomycin (50 mg/mL), rifampicin (50 mg/mL), penicillin G (50 mg/mL), and ampicillin (50 mg/mL). 

Furthermore, we confirmed the complete removal of the gut bacterial community followed by antibiotic treatment. Five *S. frugiperda* larvae of the same size were selected from each group and starved for 8 h, then sterilized with 75% alcohol for 30 s and 2% sodium hypochlorite solution for 30 s, and then washed by sterilized ddH_2_O, 1 min each time. The gut was dissected in the ultra-clean tube. The DNA from the gut samples of the four groups (SK, S, YK, and Y) was extracted, and PCR was amplified using 16S rDNA gene primers 27F and 1492R. The PCR condition involves 95 °C for 5 min, 95 °C for 30 s, 55 °C for 30 s, 72 °C for 40 s, 25 cycles, 72 °C for 7 min, and 4 °C to end. The resulting PCR products (25 cycles) were checked by electrophoresis on a 1.0% agarose gel. The absence of a visible band in the treated group (SK and YK) further confirms the removal of the gut bacterial community in *S. frugiperda* (Supplementary Figure S1).

### 2.5. Quantitative Real-Time PCR Analysis of the Immune Genes

The effects of the gut microbiome and diet on the important functions of the *S. frugiperda* were studied by analyzing the fourteen immune genes (CYP9A60, ABCC2, ABC C13-like, ABC subG1-like, GST D7-like X1, GST 2-like, UGT 1-8-like, UGT2B19-like, Gloverin-like, Attacin-A-like, Attacin-like, Cactus, LITAF, and Relish) expression patterns across the four different groups (SK, S, YK, and Y). The selection of these immune genes was based on previous studies that highlighted their important role in the defense system of insects against microbial pathogens and toxic diets. The *S. frugiperda* larvae (*n* = 5) were taken from four groups (SK, S, YK, and Y) and starved for 8 h, then sterilized with 75% alcohol for 30 s and 2% sodium hypochlorite solution for 30 s, and then washed twice in sterilized ddH_2_O for 1 min. The guts were dissected, and total RNA was extracted from the dissected midguts using the Trizol Total RNA Isolation Kit (Takara, Kusatsu, Japan). The RNA concentrations were determined by NanoDrop 2000 (Thermo Scientific, Waltham, MA, USA), and integrity was assessed on Agilent 2100 Bioanalyzer (Agilent Technologies, Santa Clara, CA, USA). The list of primers and their sequences used in RT-qPCR is shown in Table S1. RT-qPCR was carried out in a BioRad CXF96 Real-Time PCR Detection System (Bio-Rad Laboratories, Inc., Hercules, CA, USA) using TB Green^®^ Premix Ex TaqTM II (Tli RNaseH Plus) (TaKaRa) according to the instructions of the manufacturer, and the housekeeping RpL18 gene was used as a control in this experiment. The reaction program was set as initial denaturation at 95 °C for 30 s, 40 cycles of 95 °C for 5 s, and 60 °C for 30 s. The expression level of genes was calculated by the 2^–ΔΔCt^ method, and the value stood for an n-fold difference relative to the calibrator (RpL18). Each experiment was performed in triplicate. All data were given in terms of relative mRNA expression as mean ± SE.

### 2.6. Isolation of Gut Microbiota from S. frugiperda

In order to prepare dissected midguts from *S. frugiperda*, larvae were surface sterilized in 75% ethanol and rinsed twice with PBS buffer. The dissected midguts were then ground in 200 μL ddH_2_O for the isolation of bacteria. The homogenate was plated onto LB agar plates and incubated for 24 h at 30 °C. After 24 h, the bacterial colonies which showed growth were picked and re-plated. The 16S rRNA gene of the isolates was PCR amplified using universal 16S rRNA gene primers 27F and 1492R, and the resulting PCR products were sequenced by Tsingke (Guangzhou, China). The sequenced bacterial strains were then identified via BLAST search against the NCBI rRNA/ITS database (https://www.ncbi.nlm.nih.gov/ (accessed on 30 July 2022)). 

### 2.7. Removal of Gut Microbiota from S. frugiperda Using Antibiotic Treatment

Next, we reintroduced the bacterial isolates to the antibiotic-treated *S. frugiperda* larvae via oral feeding. Prior to oral feeding, all the isolated midgut bacteria were sub-cultured overnight, centrifuged, diluted (OD600 = 1.0), and mixed with the preservative-free artificial diet. Thirty larvae in each group were fed with a 30% sucrose solution containing virus (ACMNPV) for two consecutive days after 12 h of starvation. On the third day, the larvae were fed the artificial diet containing midgut bacterial isolates, and two days later, the normal diet was restored. Mortality was recorded every 12 h. The final results were analyzed using GraphPad Prism 8.0, and each experiment was replicated three times. 

### 2.8. Statistical Analysis

The difference between the bacterial communities was measured using the unweighted UniFrac approach [44]. Heatmap was used to determine the relative abundance of the top 50 bacterial genera in the samples using the imageGP online tool with log 2-transformed data [45]. Differences in the OTUs and plant biomass-degrading genes of the “lab diet” and “field diet” groups were examined using Welch’s test in the STAMP software. One-way analysis of variation (ANOVA) followed by least-significant difference (LSD) multiple comparisons were used to compare the relative expression of immune genes in different groups. Differences were considered statistically significant at *p* < 0.05.

## 3. Results

### 3.1. Illumina Sequencing Statistics

In-depth knowledge of the bacterial communities associated with the digestion of the specific agricultural plant materials present in Guangzhou, China, was assessed by the metabarcoding approach. In the present study, gut samples from the different populations of *S. frugiperda* of Guangzhou, China, reared on two different diets, were analyzed by sequencing the variable V4 regions of the 16S rRNA gene. This work represents a comprehensive analysis of the contrasting bacterial communities in two population groups of *S. frugiperda* each fed with either the artificial or the leaf diet. After the pre-processing and quality assessment of the reads, a total of 4,98,512 high-quality reads were obtained with an average length of 251 ± 1 bp. 

### 3.2. Diversity Analysis of Midgut Microbiota in “Lab Diet” and “Field Diet” Groups

**Figure 1:**
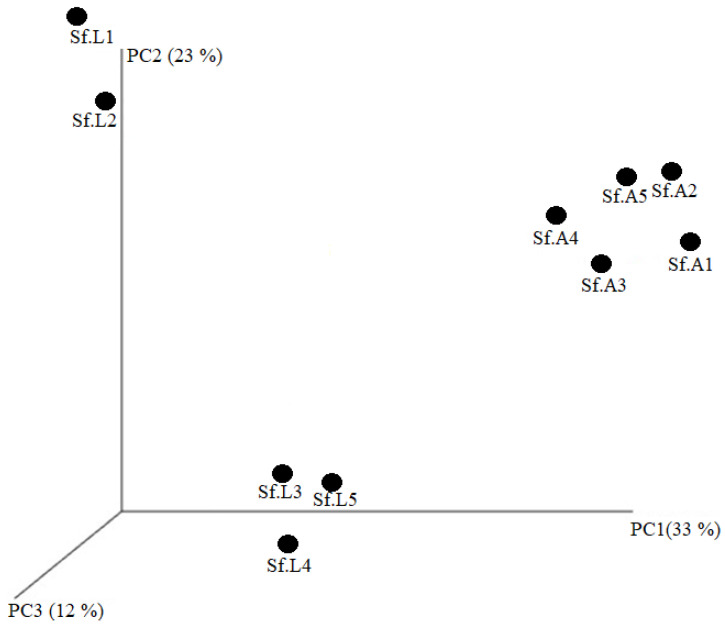
Beta diversity analysis: The PCoA showed the unweighted Unifac distance. Datasets were subsampled to equal depth before the UniFrac distance computation. *S. frugiperda* population feeding on corn leaf (Sf.L1,2,3,4,5); *S. frugiperda* population feeding on starch-rich artificial diet (Sf.A1, 2,3,4,5).

A total of 237 OTUs at a 97% threshold were generated from the pre-processed reads obtained from the ten samples. We employed three species-richness measures of observed species, Shannon’s diversity, Simpson indices, Chao1, and abundance-based coverage estimator (ACE) to investigate the number of different OTUs between the “lab diet” and “field diet” groups. The ACE values in the “lab diet” and “field diet” armyworm groups were 138.65 ± 26.67 and 88.04 ± 35.11, respectively (*p* < 0.05). The Chao1 values in the “lab diet” and “field diet” groups were 129.77 ± 21.78 and 85.19 ± 33.99, respectively (*p* < 0.05). The Shannon indices in the “lab diet” and “field diet” groups were 0.93 ± 0.10 and 0.57 ± 0.30, respectively (*p* = 0.055). The Simpson indices in the “lab diet” and “field diet” groups were 0.48 ± 0.01 and 0.73 ± 0.19, respectively (*p* < 0.05) (Table 1). The Shannon index suggested a higher diversity in bacterial populations from the “lab diet” samples than from the “field diet” samples; however, the difference is not statistically significant. Similarities of the microbial community compositions between the “lab diet” and “field diet” groups were compared by PCoA based on unweighted Unifac distance, which shows that the “lab diet” and “field diet” groups are separated. This indicates that the microbial communities in the “lab diet” and “field diet” groups have significant differences, which further reveals the difference between the bacterial communities present in the samples (Figure 1).

### 3.3. Bacterial Community of S. frugiperda Reared in “Lab Diet” and “Field Diet”

**Figure 2:**
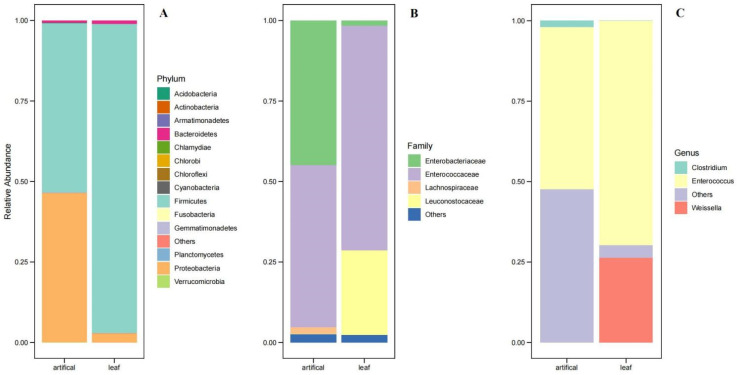
Relative abundance of bacterial community in the “lab diet” and “field diet” groups at (**A**) phylum level, (**B**) family level, (**C**) genus level. Artificial: *S. frugiperda* population feeding on lab (artificial) diet; leaf: *S. frugiperda* population feeding on field (corn leaf) diet.

Overall, the *S. frugiperda* larvae were dominated by the bacterial phyla Firmicutes and Proteobacteria. The two most abundant bacterial phyla in the “lab diet” group were Firmicutes (52.66%) and Proteobacteria (46.32%), comprising approximately 99% of the 16S rRNA gene sequences. In addition, planctomycetes, Chlorobi, Actinobacteria, OD1, NKB19, Chloroflexi, Armatimonadetes, Bacteroidetes, Chlamydiae, Acidobacteria, GN04, cyanobacteria, OP11, Gemmatimonadetes, Verrucomicrobia, and Thermi were present in the lab-reared samples, but at relatively low abundances. The “field diet” group was abundant, with the phylum Firmicute comprising approximately 96% of the 16S rRNA gene sequences. Other identified phyla present in low abundance in the “field diet” group were Proteobacteria, Chlorobi, fusobacteria, actinobacteria, NKB19, Chloroflexi, Bacteroidetes, Acidobacteria, GN04, cyanobacteria, Verrucomicrobia, and Thermi. Enterococcaceae, Enterobacteriaceae, and Leuconostocaceae were the most representative families detected in the analyzed samples. The lab diet samples were abundant with Enterococcaceae (50%) and Enterobacteriaceae (44.89%), whereas the “field diet” samples were enriched with the bacterial phyla Enterococcaceae (69.69%) and Leuconostocaceae (26.31%). Despite the difference in diet, the core community is associated with the gut microbial communities of *S. frugiperda* of Guangzhou, China, which was represented by three genera: *Enterococcus, Weissella,* and *Clostridium*. The genus *Enterococcus* was present abundantly in both the “lab diet” (50.34%) and “field diet” (69.69%) groups. Interestingly, the bacterial genera *Weissella* (26.31%) were only observed in the “field diet” samples (Figure 2A–C). The bacterial community of individual replicates of the ab diet” are shown in Supplementary Figure S2. Among the 237 identified OTUs, 4 (OTU1, OTU2, OTU3, and OTU4) were identified as dominant enriched OTUs, accounting for 97.14% of the total OTUs in the complete dataset. The most dominant phylotype, OTU1 (60.11%), was classified as *Enterococcus casseliflavus*. The second dominant OTU was OTU3 (22.66%), classified under the family Enterobacteriaceae. In contrast, the two other dominant OTU2 and OTU4 were classified under the genus *Weissella* and *Clostridium*, respectively (Figure 2A). 

#### Comparison of the Gut Microbiota of *S. frugiperda* Reared in “Lab Diet” and “Field Diet”

**Figure 3:**
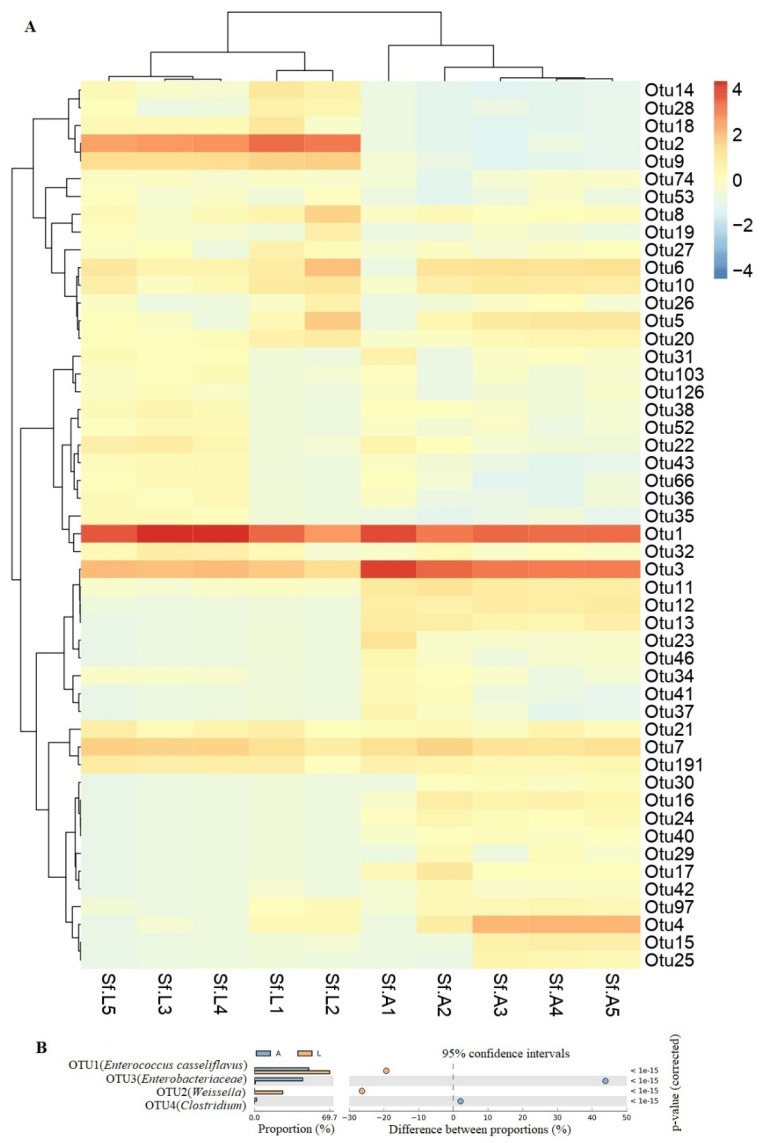
Relative abundance of the Operational taxonomic units (OTUs): (**A**) Heatmap analysis showing the relative abundance of the top 50 bacterial OTUs. *S. frugiperda* population feeding on corn leaf (Sf.L1,2,3,4,5); *S. frugiperda* population feeding on starch-rich artificial diet (Sf.A1, 2,3,4,5). (**B**) The significant difference between the abundant bacterial OTUs. A: Artificial (lab) diet; L: Leaf (field) diet.

The heatmap of the 50 most abundant OTUs shows the similarities and differences between the samples (Figure 3A). OTU1 and OTU2 were significantly higher in the field group than in the lab group, whereas OTU3 and OTU4 were higher in the lab diet group (Figure 3B). 

### 3.4. Predictions of Metabolic Potentials

The PICRUSt analysis revealed a link between gut bacteria and host metabolic changes. The significant differences in the functional properties of the ”lab diet” and “field diet” groups are shown in Supplementary Figure S3. Specifically, the pathways associated with carbohydrate metabolism, translation, nucleotide metabolism, lipid metabolism, xenobiotics biodegradation and metabolism, transcription, xenobiotic biodegradation and metabolism, and DNA replication and repair were significantly higher in the “field diet” group, whereas energy metabolism, amino acid metabolism, cellular processing, signaling, and membrane transport were significantly higher in the “lab diet” group (Supplementary Figure S3). We further identified the genes associated with the plant biomass deconstruction (lignin, hemicellulose, cellobiose, and cello-oligosaccharides) in the PICRUSt predicted metagenome. A large number of predicted genes that codify for the plant biomass-degrading enzymes were present in both groups with varying abundance. The genes coding for the enzymes glyceraldehyde 3-phosphate dehydrogenase [EC:1.2.1.12]; beta-galactosidase [EC:3.2.1.23]; alpha-galactosidase [EC:3.2.1.22]; evolved beta-galactosidase subunit alpha [EC:3.2.1.23]; alpha-mannosidase [EC:3.2.1.24]; carboxylesterase [EC:3.1.1.1]; thiol peroxidase, atypical 2-Cys peroxiredoxin [EC:1.11.1.15]; beta-fructofuranosidase [EC:3.2.1.26] were present in high abundance in the “field diet” samples compared to the “lab diet” samples (Supplementary Figure S4).

### 3.5. Effect of the Gut Microbiome on the Host Immune Gene Expression

**Figure 4:**
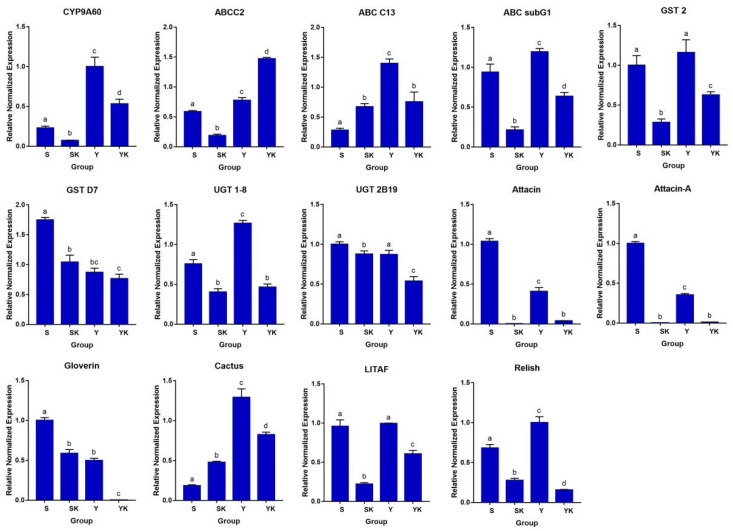
Relative expression levels of immunity-related genes in four different groups of *S. frugiperda* (S, SK, Y, and YK). Error bars represent mean ± SD from three independent experiments. The same letters above bars indicate no significant difference (*p* ≥ 0.05).

Quantitative real-time PCR was used to evaluate the impact of gut microbiota depletion on the expression levels of 14 immune-related genes (CYP9A60, ABCC2, ABC C13-like (ABC C13), ABC subG1-like (ABC subG1), GST 2-like (GST2), GST D7-like X1 (GST D7), UGT 1-8-like (UGT 1-8), UGT2B19-like (UGT 2B19), Attacin-A-like(Attacin-A), Attacin-like (Attacin), Gloverin-like (Gloverin), Cactus, LITAF, and Relish) of *S. frugiperda*. The results illustrate that most of the studied immune genes were significantly downregulated in antibiotic-treated *S. frugiperda* (SK and YK). The expression level of CYP9A60, ABC subG1, GST D7, GST 2, UGT 1-8, UGT2B19, Gloverin, Attacin-A, Attacin, LITAF, and Relish were significantly lower (*p* < 0.05) in the antibiotic treatment groups (SK and YK) than those in the untreated groups (S and Y). However, an upregulated expression (*p* < 0.05) of ABCC2 in the YR group and of ABCC13 and Cactus in the SK group were observed (Figure 4).

### 3.6. Isolation of Midgut Microbiota from S. frugiperda

A selective medium was used to cultivate and identify the specific midgut bacteria, and seven bacterial species were identified from the midgut of *S. frugiperda* larvae (Table S2). The 16S rDNA gene sequence of each of the isolated strains was blasted with the sequence published in GenBank (Table S2); our results show that two isolated midgut bacteria from both the lab diet (SfS) and the field diet (SfY) groups are the same bacteria, namely SfY4 (SfS1) and SfY5 (SfS2). Additionally, the closest relatives were selected to construct the phylogenetic tree (Supplementary Figure S5). The tree showed that the isolates were phylogenetically placed within four clades and that SfS1, SfS2, SfY4, and SfY5 closely belonged to the *Enterococcus* clade, while SfY3 closely belonged to the *Bacillus* clade, SfY1 closely belonged to the *Klebsiella* clade, and SfY2 closely belonged to the *Microbacterium* clade. 

### 3.7. Effect of Gut Microbiota on Autographa Californica Nucleopolyhedrovirus (AcMNPV) Pathogenesis in S. frugiperda Larvae

**Figure 5:**
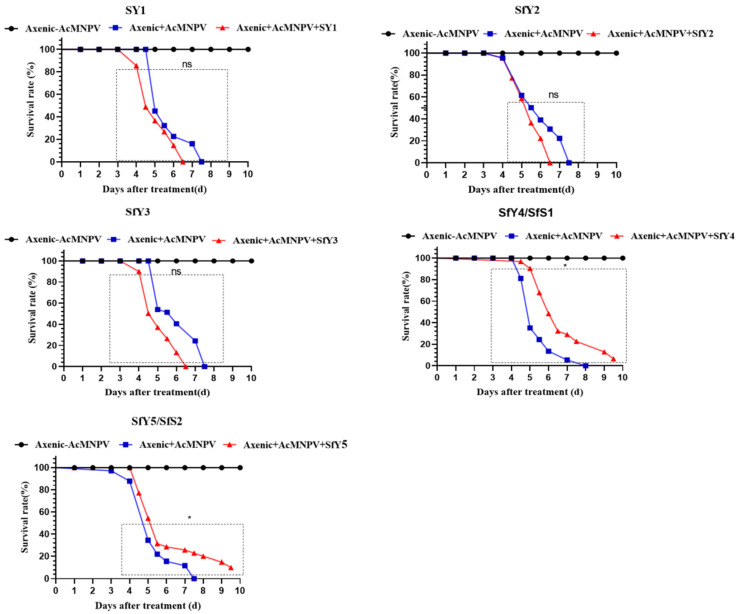
Effect of five isolated gut bacteria (SfY1, SfY2, SfY3, SfY4 (SfS1), and SfY5 (SfS2)) on AcMNPV pathogenesis in *S. frugiperda* larvae (*n* = 30). Three biological replicates were conducted, and the significance was determined by log-rank (Mantel-Cox) test. Asterisk indicates statistically significant (*p* < 0.01), and ns indicates no significant difference.

Axenic larvae of *S. frugiperda* were prepared by oral antibiotic treatment to investigate the possible role of the gut microbiota in the pathogenesis of AcMNPV in S. frugiperda. The oral reintroduction of SfY4 (SfS1) (*E. mundtii*) and SfY5 (SfS2) (*E. gallinarum*) into antibiotic-treated *S. frugiperda* significantly reduced host susceptibility to AcMNPV treatment (*p* <0.01, log-rank (Mantel-Cox) test). In contrast, three other commensal bacteria, namely SfY1 (*K. variicola*), SfY2 (*M. hatanonis*), and SfY3 (*B. paramycoides*) had a positive effect on AcMNPV toxicity, which decreased the median survival of antibiotic-treated *S. frugiperda* (*p* > 0.01, log-rank (Mantel-Cox) test) (Figure 5). 

## 4. Discussion

In the present study, we analyzed the gut bacterial communities and their imputed function in *S. frugiperda* feeding on a corn leaf and a starch-rich artificial diet. Comparing two different bacterial communities illustrates their specific bacterial members and their functional activities with the particular diet, including the plant biomass grown in this region. This study is the first to employ 16S rRNA Illumina HiSeq technology to compare differences in the gut microbiota between “lab diet” and “field diet” groups of *S. frugiperda.*

Compared to other insects, the digestive system of Lepidoptera is unique due to its high alkaline pH (pH > 10) which limits the growth of specialized species that can survive in these extreme environments [46,47]. Moreover, diet may also profoundly affect microbial diversity and communities. In the present study, the microbial diversity was higher in “lab diet” insects compared to “field diet” insects. This may also indicate that the presence of unique, specialized microorganisms in the gut of the “field diet” group may be associated with the digestion of the corn plant materials. On the other hand, the artificial diet having various ingredients may facilitate the growth of diverse bacterial members in the gut microbiota of the *S. frugiperda.* Analysis of beta diversity further supports the differences among the bacterial communities in the studied samples. In brief, our results suggest a variation in the gut microbial community structure of *S. frugiperda* reared using two different diets. Our study is consistent with previous studies, which showed that diet could affect the bacterial community structure in many insect species [48,49,50]

Overall, our results reveal the presence of a relatively high abundance of bacterial phyla Firmicutes and Proteobacteria in the gut samples of *S. frugiperda* collected from Guangzhou, China. This observation is consistent with the previous reports on *S. frugiperda* collected from Nigeria [37] and Kenya [36]. The high abundance of these bacterial phyla have also been reported in other lepidopteran insects such as *Plutella xylostella* [51], *Busseola fusca* [52], *Choristoneura fumiferana* [53], *Ostrinia nubilalis* [54], etc. Our study shows that the relative abundance of Firmicutes was higher in the field diet group compared to the lab diet group. The members under this phyla are known to participate in the energy absorption from the diet, including the degradation of cellulose and hemicellulose and the metabolism of various amino acids [55,56,57]. This may be due to the presence of high cellulose and hemicellulose contents in the leaf samples, which increases the need for more Firmicutes to digest the food [58].

Analysis of the bacterial community reveals that the major identified families were Enterococcaceae, Enterobacteriaceae, and Leuconostocaceae. The presence of the family Leuconostocaceae was significantly higher in the “field diet” samples, whereas lab diet samples exhibited an abundance of the Enterobacteriaceae family. The members of this family are Gram-positive, non-spore-forming bacteria, usually present in nutrient-rich environments, including vegetable waste, and actively involved in heterofermentative carbohydrate metabolism [59,60]. This phylum is also reported in the larval gut of wood-feeding beetle [61]. In comparison, most of the members under the Enterobacteriaceae family are symbionts and were suggested to facilitate host digestive activities within the gut [62]. 

However, the core bacterial community was dominated by the bacterial genus *Enterococcus* in both the “lab diet” and the “field diet” groups. They are commonly found in the gut of Lepidopteran species in wild and laboratory-reared conditions [63,64]. The highest dominant OTU1 classified as *Enterococcus casseliflavus* was present in a significantly higher number in the “field diet” samples when compared to the “lab diet” samples. The presence of the bacterial genus *Enterococcus* is in line with earlier reports on *S. frugiperda* collected from Brazil [34], Kenya [36], Nigeria [37], and other Lepidoptera, such as *Spodoptera litura* [65], *Manduca sexta* [66], *Hyles euphorbiae* [28], *Helicoverpa armigera* [67], *Heliothis virescens* [68], etc. Research also showed that the *E. casseliflavus* present in the gut of *S. litura* is able to crystalize some toxic compounds rich in terpenes (in particular α- carotenoids and β-carotenoids produced by *Phaseolus lunatus* (lima beans) [65]) and that *M. sexta* feed on toxic Solanaceae [66]. *E. casseliflavus* isolates from *Hyles euphorbiae* also were also suggested to play an important role in the immobilization of toxic molecules [28]. This bacterial species has previously been isolated from insecticide (chlorpyrifos ethyl, lambda-cyhalothrin, deltamethrin, and spinosad)-resistant strains of *S. frugiperda* [34]. These observations indicate that the identified OTU1 might play a significant role in detoxifying plant materials in different Lepidoptera species, including *S. frugiperda.* However, further works need to be carried out to illustrate the role of OTU1 in the pathogenicity of *S. frugiperda,* especially for the damage to the corn leaf. The second dominant phylotype OTU2, classified as *Weissella,* was significantly more present in the “field diet” larvae. The members under this genus are Gram-positive lactic acid bacteria that are non-spore-forming, nonmotile, heterofermentative, catalase-negative, and short rod-shaped, and they possess anti-cancer, anti-inflammatory, antibacterial, anti-fungal, and immune-boosting potential while being extensively used for the preparation of fermented foods as well as in probiotics [69,70,71,72]. In contrast to our study, Weissella has not been reported as a dominant bacterial genus of *S. frugiperda* in other countries such as Brazil, Kenya, and Nigeria [34]. Although they have been previously detected in several insects such as *Cryptocercus kyebangensis* [73] or bumblebees [74], their specific role in the insect physiology is not yet revealed. The third and fourth dominant OTU were identified as *Enterobacteriaceae* and *Clostridium*, respectively. The genus *Clostridium* is commonly present in the anoxic gut atmosphere of lepidopteran insects and could be involved in the degradation of amino acids in the laboratory diet group [57,75].

PICRUSt may indicate important clues for predicting the functional profiles of the gut bacterial community. The result suggests that the fall armyworm was enriched with the microbial genes involved in the metabolism of carbohydrate, energy metabolism, lipid metabolism, xenobiotics biodegradation, etc., which may be explained by their intensive feeding on the carbohydrate-rich diet as well as corn leaf. However, the relative abundance of the predicted genes in the “lab diet” and “field diet” groups also differ significantly. Further analysis identified several genes that codify for the plant biomass-degrading enzymes, which indicates the ability of the natural gut microbiota of *S. frugiperda* to damage plant materials. However, a significant difference in the relative abundance of these genes might be due to the selection pressure in the “lab diet” and “field diet” populations that favors the enzymatic activities associated with the degradation of plant complex polysaccharides. However, further studies, such as shotgun metagenomics or functional assays, should be performed to validate the PICRUSt predicted outputs obtained from this study.

The insect gut microbiota plays an important role in host health by modulating the host’s immune system [76]. In the present study, the removal of gut microbiota using antibiotic treatment was performed to verify whether gut microbiota has any effect on the immune gene expression in *S. frugiperda*. The results indicate that most of the immune genes are significantly down-regulated in the midgut of axenic *S. frugiperda*, suggesting that the presence of commensal bacteria could trigger a basal level of immunity, leading to enhanced AMPs expression. These findings are in line with results reported by other publications in the literature that have shown that a disruption of the gut microbiota by antibiotics reduces the immune response of insect larvae [76,77]. It has also been reported that several biological processes, such as energy production, metabolism, and the autophagy–lysosome signal pathway, were affected after antibiotics-induced dysbiosis [78,79,80]. The induction of the host immune response by the gut microbiota is also crucial for regulating other microbiota, including pathogens [76]. Diet composition has a profound effect on insect immunity [81,82]. The present study observed that most of the immune gene expression was higher in the insects reared in the field diet compared to the lab diet. This led to the suggestion that the presence of various microbes in the field diet may interact with the microbial symbionts and enhance the immune response in the host. It is also possible that toxic compounds present in field diets may deplete the gut microbial community, leading to the overgrowth of host pathogens and thereby enhancing host immunity [83]. Toxic compounds have also been shown to alter immune gene expression and antioxidant-mediated defense responses in insects [84]. 

Insect gut microbiota plays an important role in the defense against various pathogens [85]. In the present study, the role of the gut microbiota in the pathogenesis of *AcMNPV* in *S. frugiperda* was tested by reintroducing gut bacterial isolates to AcMNPV-treated axenic insects. The oral reintroduction of gut bacterial isolates (*E. mundtii* and *E. gallinarum*) significantly enhances host resistance to pathogen infection. These results are consistent with Sun et al. (2016), who demonstrated that the abundance of the bacterial genus *Enterococcus* is associated with increased resistance to insects [86]. It is believed that insect gut microbes enhance host defenses by priming the host’s immune system or by supplementing nutrients for host metabolic homeostasis [87,88,89]. Taken together, our results indicate that diet composition influences the host microbiome and immune response of *S. frugiperda*, ultimately playing a key role in the pest defense system and its fitness in the natural environment. 

Bacterial symbionts play a major role in host growth, development, and metabolism. They are known to be associated with gene expression and relevant metabolic pathways (fatty acid metabolism, epidermal growth, detoxification, etc.) involved in host development. Therefore, the effects of antibiotics on growth, development, and reproduction of *S. frugiperda* and, more specifically, the roles of key symbionts in the host developmental process need to be addressed in the future [90,91]. The gut microbiota is also known to be involved in bi-directional communication with host circadian rhythms and their composition can vary at different time points [92]. However, the present study primarily focused on the gut microbiota of the third instar larval stage at a single time point and did not explore microbial populations at other developmental stages. Therefore, future work needs to focus on identifying the optimal sampling time by analyzing the insect gut microbiome at different time intervals to avoid or minimize the loss of any specific microbiota during analysis. 

## 5. Conclusions

This study provides novel information regarding the gut bacterial diversity of *S. frugiperda,* demonstrating the bacterial communities of larvae that were reared on an artificial and natural (corn leaf) diet. We conclude that a significant difference is evident in the bacterial communities among the “lab diet” and “field diet” group; both the “lab diet” and “field diet” group were abundant with the bacterial phyla Firmicutes and Proteobacteria; only a few phylotypes are dominated in the bacterial community; PICRUSt predicted functional analysis indicates the presence of several genes associated with the plant’s biomass degradation. Overall, our findings indicate that diet composition influences the insect gut microbiome, which affects the defense system of *S. frugiperda*.

## Figures and Tables

**Table 1:** Comparison of α-diversity indices of gut microbiota from the “lab diet” and “field diet” groups *.

Alpha Diversity Index	Lab Diet	Field Diet	*p*-Value
Observed OTUs	108.6 ± 11.43	75.80 ± 31.01	0.09
Chao	129.77 ± 21.78	85.19 ± 33.99	0.03
Ace	138.65 ± 26.67	88.04 ± 35.11	0.01
Shannon	0.93 ± 0.10	0.57 ± 0.30	0.05
Simpson	0.48 ± 0.01	0.73 ± 0.19	0.03
Coverage	0.999 ± 0.0001	0.999 ± 0.0001	-

* Welch’s *t*-test was used to compare the Alpha diversity index between lab diet and field diet groups using STAMP software.

## Data Availability

The datasets generated for this study are accessible upon request from the authors.

## Supplementary Materials

The following supporting information can be downloaded at: https://www.mdpi.com/article/10.3390/biology11111602/s1. Figure S1: PCR amplification of the 16S rDNA gene. YK: field diet with antibiotics, Y: field diet without; Figure S2: antibiotics; SK: lab diet with antibiotics, S: lab diet without antibiotics; Relative abundance of the bacterial community at the phylum level. *S. frugiperda* population feeding on corn leaf (Sf.L1,2,3,4,5); *S. frugiperda* population feeding on starch-rich artificial diet (Sf.A1, 2,3,4,5); Figure S3: Inferred functions of bacterial communities associated with S. frugiperda. All of the predicted KEGG metabolic pathways are shown at the second hierarchical level and grouped by major functional categories. Field diet group: *S. frugiperda* population feeding on field diet (corn leaf); Lab diet group: *S. frugiperda* population feeding on lab diet (starch-rich artificial diet); Figure S4: Inferred functions of bacterial communities associated with the plant biomass-degrading gene. Field diet group: *S. frugiperda* population feeding on field diet (corn leaf); Lab diet group: *S. frugiperda* population feeding on lab diet (starch-rich artificial diet); Figure S5: Phylogenetic analysis of seven isolates with related bacteria strains based on 16S rDNA sequence. Phylogeny was estimated by using the neighbor-joining method conducted in MEGA7 software. The scale 0.020 is the genetic distance. Bootstrap values (in percent) are based on 1000 replications. Sequence GenBank accession numbers are shown in close to clade position; Table S1: List of Primers and their sequences used in RT-qPCR; Table S2: The summary of 16S rRNA blast results of cultivable bacteria isolates.

## Abbreviations

PICRUSt	Phylogenetic Investigation of Communities by Reconstruction of Unobserved States
HTS	High throughput sequencing
OTUs	Operational taxonomic units
ACE	Abundance-based coverage estimator

## References

[1] Douglas A.E. (2015). Multiorganismal insects: Diversity and function of resident microorganisms. Annu. Rev. Entomol..

[2] Yek S.H., Mueller U.G. (2011). The metapleural gland of ants. Biol. Rev..

[3] Ruokolainen L., Ikonen S., Makkonen H., Hanski I. (2016). Larval growth rate is associated with the composition of the gut microbiota in the Glanville fritillary butterfly. Oecologia.

[4] Leitão-Gonçalves R., Carvalho-Santos Z., Francisco A.P., Fioreze G.T., Anjos M., Baltazar C., Elias A.P., Itskov P.M., Piper M.D.W., Ribeiro C. (2017). Commensal bacteria and essential amino acids control food choice behavior and reproduction. PLoS Biol..

[5] Ankrah N.Y.D., Douglas A.E. (2018). Nutrient factories: Metabolic function of beneficial microorganisms associated with insects. Environ. Microbiol..

[6] Shao Y., Chen B., Sun C., Ishida K., Hertweck C., Boland W. (2017). Symbiont-derived antimicrobials contribute to the control of the Lepidopteran gut microbiota. Cell Chem. Biol..

[7] Jones A.G., Mason C.J., Felton G.W., Hoover K. (2019). Host plant and population source drive diversity of microbial gut communities in two polyphagous insects. Sci. Rep..

[8] Broderick N.A., Raffa K.F., Handelsman J. (2006). Midgut bacteria required for Bacillus thuringiensis insecticidal activity. Proc. Natl. Acad. Sci. USA.

[9] Pinto-Tomás A., Uribe-Lorío L., Blanco J., Fontecha G., Rodríguez C., Mora M., Janzen D., Chavarría F., Díaz J., Sittenfeld A. (2007). Actividades enzimáticas en aislamientos bacterianos de tractos digestivos de larvas y del contenido de pupas de Automeris zugana y Rothschildia lebeau (Lepidoptera: Saturniidae). Rev. De Biol. Trop..

[10] Prem Anand A.A., Vennison S.J., Sankar S.G., Gilwax Prabhu D.I., Vasan P.T., Raghuraman T., Jerome Geoffrey C., Vendan S.E. (2010). Isolation and characterization of bacteria from the gut of Bombyx mori that degrade cellulose, xylan, pectin and starch and their impact on digestion. J. Insect Sci..

[11] Wang J., Peiffer M., Hoover K., Rosa C., Zeng R., Felton G.W. (2017). Helicoverpa zea gut-associated bacteria indirectly induce defenses in tomato by triggering a salivary elicitor(s). New Phytol..

[12] Hammer T.J., Janzen D.H., Hallwachs W., Jaffe S.P., Fierer N. (2017). Caterpillars lack a resident gut microbiome. Proc. Natl. Acad. Sci. USA.

[13] Sparks A.N. (1979). A review of the biology of the fall armyworm. Fla. Entomol..

[14] Montezano D.G., Specht A., Sosa-Gómez D.R., Roque-Specht V.F., Sousa-Silva J.C., Paula-Moraes S.V.d., Peterson J.A., Hunt T. (2018). Host plants of Spodoptera frugiperda (Lepidoptera: Noctuidae) in the Americas. Afr. Entomol..

[15] Feldmann F., Rieckmann U., Winter S. (2019). The spread of the fall armyworm Spodoptera frugiperda in Africa—What should be done next?. J. Plant Dis. Prot..

[16] Goergen G., Kumar P.L., Sankung S.B., Togola A., Tamò M. (2016). First report of outbreaks of the fall armyworm Spodoptera frugiperda (JE Smith)(Lepidoptera, Noctuidae), a new alien invasive pest in West and Central Africa. PLoS ONE.

[17] Sharanabasappa, Kalleshwaraswamy C.M., Asokan R., Swamy H.M.M., Maruthi M.S., Pavithra H.B., Hegbe K., Navi S., Prabhu S.T., Goergen G.E. (2018). First report of the fall armyworm, Spodoptera frugiperda (JE Smith)(Lepidoptera: Noctuidae), an alien invasive pest on maize in India. Pest Manag. Hortic. Ecosyst..

[18] Guo J., Zhao J., He K., Zhang F., Wang Z. (2018). Potential invasion of the crop-devastating insect pest fall armyworm Spodoptera frugiperda to China. Plant Prot..

[19] Sharanabasappa, Kalleshwaraswamy C.M., Maruthi M.S., Pavithra H.B. (2018). Biology of invasive fall army worm Spodoptera frugiperda (JE Smith)(Lepidoptera: Noctuidae) on maize. Indian J. Entomol..

[20] Sun X.-x., Hu C.-x., Jia H.-r., Wu Q.-l., Shen X.-j., Zhao S.-y., Jiang Y.-y., Wu K.-m. (2020). Case study on the first immigration of fall armyworm Spodoptera frugiperda invading into China. J. Integr. Agric..

[21] De Groote H., Kimenju S.C., Munyua B., Palmas S., Kassie M., Bruce A. (2020). Spread and impact of fall armyworm (Spodoptera frugiperda JE Smith) in maize production areas of Kenya. Agric. Ecosyst. Environ..

[22] Bartlett A.C., Staten R.T. (1996). The Sterile Insect Release Method and Other Genetic Control Strategies. Radcliffe’s IPM World Textbook.

[23] Wright R.J. (1984). Evaluation of crop rotation for control of Colorado potato beetles (Coleoptera: Chrysomelidae) in commercial potato fields on Long Island. J. Econ. Entomol..

[24] Jakka S.R.K., Gong L., Hasler J., Banerjee R., Sheets J.J., Narva K., Blanco C.A., Jurat-Fuentes J.L. (2016). Field-evolved mode 1 resistance of the fall armyworm to transgenic Cry1Fa-expressing corn associated with reduced Cry1Fa toxin binding and midgut alkaline phosphatase expression. Appl. Environ. Microbiol..

[25] Banerjee R., Hasler J., Meagher R., Nagoshi R., Hietala L., Huang F., Narva K., Jurat-Fuentes J.L. (2017). Mechanism and DNA-based detection of field-evolved resistance to transgenic Bt corn in fall armyworm (Spodoptera frugiperda). Sci. Rep..

[26] Arora A.K., Douglas A.E. (2017). Hype or opportunity? Using microbial symbionts in novel strategies for insect pest control. J. Insect Physiol..

[27] Ceja-Navarro J.A., Karaoz U., Bill M., Hao Z., White R.A., Arellano A., Ramanculova L., Filley T.R., Berry T.D., Conrad M.E. (2019). Gut anatomical properties and microbial functional assembly promote lignocellulose deconstruction and colony subsistence of a wood-feeding beetle. Nat. Microbiol..

[28] Vilanova C., Baixeras J., Latorre A., Porcar M. (2016). The generalist inside the specialist: Gut bacterial communities of two insect species feeding on toxic plants are dominated by Enterococcus sp.. Front. Microbiol..

[29] Ceja-Navarro J.A., Vega F.E., Karaoz U., Hao Z., Jenkins S., Lim H.C., Kosina P., Infante F., Northen T.R., Brodie E.L. (2015). Gut microbiota mediate caffeine detoxification in the primary insect pest of coffee. Nat. Commun..

[30] Fadrosh D.W., Ma B., Gajer P., Sengamalay N., Ott S., Brotman R.M., Ravel J. (2014). An improved dual-indexing approach for multiplexed 16S rRNA gene sequencing on the Illumina MiSeq platform. Microbiome.

[31] Lv D., Liu X., Dong Y., Yan Z., Zhang X., Wang P., Yuan X., Li Y. (2021). Comparison of Gut Bacterial Communities of Fall Armyworm (Spodoptera frugiperda) Reared on Different Host Plants. Int. J. Mol. Sci..

[32] Chen Y.-P., Li Y.-H., Sun Z.-X., Du E.W., Lu Z.-H., Li H., Gui F.-R. (2022). Effects of Host Plants on Bacterial Community Structure in Larvae Midgut of Spodoptera frugiperda. Insects.

[33] Acevedo F.E., Peiffer M., Tan C.-W., Stanley B.A., Stanley A., Wang J., Jones A.G., Hoover K., Rosa C., Luthe D. (2017). Fall armyworm-associated gut bacteria modulate plant defense responses. Mol. Plant-Microbe Interact..

[34] Almeida L.G.d., Moraes L.A.B.d., Trigo J.R., Omoto C., Consoli F.L. (2017). The gut microbiota of insecticide-resistant insects houses insecticide-degrading bacteria: A potential source for biotechnological exploitation. PLoS ONE.

[35] Rozadilla G., Cabrera N.A., Virla E.G., Greco N.M., McCarthy C.B. (2020). Gut microbiota of Spodoptera frugiperda (JE Smith) larvae as revealed by metatranscriptomic analysis. J. Appl. Entomol..

[36] Gichuhi J., Sevgan S., Khamis F., Van den Berg J., du Plessis H., Ekesi S., Herren J.K. (2020). Diversity of fall armyworm, Spodoptera frugiperda and their gut bacterial community in Kenya. PeerJ.

[37] Ugwu J.A., Liu M., Sun H., Asiegbu F.O. (2020). Microbiome of the larvae of Spodoptera frugiperda (JE Smith)(Lepidoptera: Noctuidae) from maize plants. J. Appl. Entomol..

[38] Andrews S. (2010). FastQC: A Quality Control Tool for High Throughput Sequence Data. Babraham Bioinformatics.

[39] De Mandal S., Chatterjee R., Kumar N.S. (2017). Dominant bacterial phyla in caves and their predicted functional roles in C and N cycle. BMC Microbiol..

[40] De Mandal S., Panda A.K., Bisht S.S., Senthil Kumar N. (2016). MiSeq HV4 16S rRNA gene analysis of bacterial community composition among the cave sediments of Indo-Burma biodiversity hotspot. Environ. Sci. Pollut. Res..

[41] Edgar R.C. (2013). UPARSE: Highly accurate OTU sequences from microbial amplicon reads. Nat. Methods.

[42] Edgar R.C., Haas B.J., Clemente J.C., Quince C., Knight R. (2011). UCHIME improves sensitivity and speed of chimera detection. Bioinformatics.

[43] Langille M.G., Zaneveld J., Caporaso J.G., McDonald D., Knights D., Reyes J.A., Clemente J.C., Burkepile D.E., Thurber R.L.V., Knight R. (2013). Predictive functional profiling of microbial communities using 16S rRNA marker gene sequences. Nat. Biotechnol..

[44] Lozupone C., Knight R. (2005). UniFrac: A new phylogenetic method for comparing microbial communities. Appl. Environ. Microbiol..

[45] Chen T., Liu Y.-X., Huang L. (2022). ImageGP: An easy-to-use data visualization web server for scientific researchers. iMeta.

[46] Appel H.M., Martin M.M. (1990). Gut redox conditions in herbivorous lepidopteran larvae. J. Chem. Ecol..

[47] Harrison J.F. (2001). Insect acid-base physiology. Annu. Rev. Entomol..

[48] Colman D.R., Toolson E.C., Takacs-Vesbach C.D. (2012). Do diet and taxonomy influence insect gut bacterial communities?. Mol. Ecol..

[49] Mikaelyan A., Dietrich C., Köhler T., Poulsen M., Sillam-Dussès D., Brune A. (2015). Diet is the primary determinant of bacterial community structure in the guts of higher termites. Mol. Ecol..

[50] Sugio A., Dubreuil G., Giron D., Simon J.-C. (2015). Plant–insect interactions under bacterial influence: Ecological implications and underlying mechanisms. J. Exp. Bot..

[51] Xia X., Zheng D., Zhong H., Qin B., Gurr G.M., Vasseur L., Lin H., Bai J., He W., You M. (2013). DNA sequencing reveals the midgut microbiota of diamondback moth, *Plutella xylostella* (L.) and a possible relationship with insecticide resistance. PLoS ONE.

[52] Snyman M., Gupta A.K., Bezuidenhout C.C., Claassens S., Van den Berg J. (2016). Gut microbiota of Busseola fusca (Lepidoptera: Noctuidae). World J. Microbiol. Biotechnol..

[53] Landry M., Comeau A.M., Derome N., Cusson M., Levesque R.C. (2015). Composition of the spruce budworm (Choristoneura fumiferana) midgut microbiota as affected by rearing conditions. PLoS ONE.

[54] Belda E., Pedrola L., Peretó J., Martínez-Blanch J.F., Montagud A., Navarro E., Urchueguía J., Ramón D., Moya A., Porcar M. (2011). Microbial diversity in the midguts of field and lab-reared populations of the European corn borer Ostrinia nubilalis. PLoS ONE.

[55] Li Y., Hu X., Yang S., Zhou J., Zhang T., Qi L., Sun X., Fan M., Xu S., Cha M. (2017). Comparative analysis of the gut microbiota composition between captive and wild forest musk deer. Front. Microbiol..

[56] Chen B., Teh B.-S., Sun C., Hu S., Lu X., Boland W., Shao Y. (2016). Biodiversity and activity of the gut microbiota across the life history of the insect herbivore Spodoptera littoralis. Sci. Rep..

[57] Fonknechten N., Chaussonnerie S., Tricot S., Lajus A., Andreesen J.R., Perchat N., Pelletier E., Gouyvenoux M., Barbe V., Salanoubat M. (2010). Clostridium sticklandii, a specialist in amino acid degradation: Revisiting its metabolism through its genome sequence. BMC Genom..

[58] Lü J., Guo W., Chen S., Guo M., Qiu B., Yang C., Lian T., Pan H. (2019). Host plants influence the composition of the gut bacteria in Henosepilachna vigintioctopunctata. PLoS ONE.

[59] Björkroth J., Holzapfel W., Dworkin M. (2006). Genera Leuconostoc, Oenococcus and Weissella. The Prokaryotes: A Handbook on the Biology of Bacteria: Firmicutes, Cyanobacteria.

[60] Chelo I.M., Ze-Ze L., Tenreiro R. (2007). Congruence of evolutionary relationships inside the Leuconostoc–Oenococcus–Weissella clade assessed by phylogenetic analysis of the 16S rRNA gene, dnaA, gyrB, rpoC and dnaK. Int. J. Syst. Evol. Microbiol..

[61] Scully E.D., Geib S.M., Hoover K., Tien M., Tringe S.G., Barry K.W., del Rio T.G., Chovatia M., Herr J.R., Carlson J.E. (2013). Metagenomic profiling reveals lignocellulose degrading system in a microbial community associated with a wood-feeding beetle. PLoS ONE.

[62] Mereghetti V., Chouaia B., Montagna M. (2017). New Insights into the Microbiota of Moth Pests. Int. J. Mol. Sci..

[63] Broderick N.A., Raffa K.F., Goodman R.M., Handelsman J. (2004). Census of the bacterial community of the gypsy moth larval midgut by using culturing and culture-independent methods. Appl. Environ. Microbiol..

[64] Shao Y., Arias-Cordero E., Guo H., Bartram S., Boland W. (2014). In vivo Pyro-SIP assessing active gut microbiota of the cotton leafworm, Spodoptera littoralis. PLoS ONE.

[65] Shao Y., Spiteller D., Tang X., Ping L., Colesie C., Münchberg U., Bartram S., Schneider B., Büdel B., Popp J. (2011). Crystallization of α-and β-carotene in the foregut of Spodoptera larvae feeding on a toxic food plant. Insect Biochem. Mol. Biol..

[66] Brinkmann N., Martens R., Tebbe C.C. (2008). Origin and diversity of metabolically active gut bacteria from laboratory-bred larvae of Manduca sexta (Sphingidae, Lepidoptera, Insecta). Appl. Environ. Microbiol..

[67] Madhusudan S., Jalali S., Venkatesan T., Lalitha Y., Srinivas R. (2011). 16S rRNA gene based identification of gut bacteria from laboratory and wild larvae of Helicoverpa armigera (Lepidoptera: Noctuidae) from tomato farm. Bioscan.

[68] Staudacher H., Kaltenpoth M., Breeuwer J.A., Menken S.B., Heckel D.G., Groot A.T. (2016). Variability of bacterial communities in the moth Heliothis virescens indicates transient association with the host. PLoS ONE.

[69] Srionnual S., Yanagida F., Lin L.-H., Hsiao K.-N., Chen Y.-s. (2007). Weissellicin 110, a newly discovered bacteriocin from Weissella cibaria 110, isolated from plaa-som, a fermented fish product from Thailand. Appl. Environ. Microbiol..

[70] Lee W., Cho S.-M., Kim M., Ko Y.-G., Yong D., Lee K. (2013). Weissella confusa bacteremia in an immune-competent patient with underlying intramural hematomas of the aorta. Ann. Lab. Med..

[71] Kamboj K., Vasquez A., Balada-Llasat J.-M. (2015). Identification and significance of Weissella species infections. Front. Microbiol..

[72] Kang M.-S., Yeu J.-E., Hong S.-P. (2019). Safety Evaluation of Oral Care Probiotics Weissella cibaria CMU and CMS1 by Phenotypic and Genotypic Analysis. Int. J. Mol. Sci..

[73] Heo J., Hamada M., Cho H., Weon H.-Y., Kim J.-S., Hong S.-B., Kim S.-J., Kwon S.-W. (2019). Weissella cryptocerci sp. nov., isolated from gut of the insect Cryptocercus kyebangensis. Int. J. Syst. Evol. Microbiol..

[74] Praet J., Meeus I., Cnockaert M., Houf K., Smagghe G., Vandamme P. (2015). Novel lactic acid bacteria isolated from the bumble bee gut: Convivina intestini gen. nov., sp. nov., Lactobacillus bombicola sp. nov., and Weissella bombi sp. nov. Antonie Van Leeuwenhoek.

[75] Tang X., Freitak D., Vogel H., Ping L., Shao Y., Cordero E.A., Andersen G., Westermann M., Heckel D.G., Boland W. (2012). Complexity and variability of gut commensal microbiota in polyphagous lepidopteran larvae. PLoS ONE.

[76] Kwong W.K., Mancenido A.L., Moran N.A. (2017). Immune system stimulation by the native gut microbiota of honey bees. R. Soc. Open Sci..

[77] Duan X., Zhao B.a., Jin X., Cheng X., Huang S., Li J. (2021). Antibiotic Treatment Decrease the Fitness of Honeybee (Apis mellifera) Larvae. Insects.

[78] Chen Y., Zhou H., Lai Y., Chen Q., Yu X.-Q., Wang X. (2021). Gut Microbiota Dysbiosis Influences Metabolic Homeostasis in Spodoptera frugiperda. Front. Microbiol..

[79] Li G., Xia X., Zhao S., Shi M., Liu F., Zhu Y. (2020). The physiological and toxicological effects of antibiotics on an interspecies insect model. Chemosphere.

[80] Thakur A., Dhammi P., Saini H.S., Kaur S. (2016). Effect of antibiotic on survival and development of Spodoptera litura (Lepidoptera: Noctuidae) and its gut microbial diversity. Bull. Entomol. Res..

[81] Krams I.A., Kecko S., Jõers P., Trakimas G., Elferts D., Krams R., Luoto S., Rantala M.J., Inashkina I., Gudrā D. (2017). Microbiome symbionts and diet diversity incur costs on the immune system of insect larvae. J. Exp. Biol..

[82] Fellous S., Lazzaro B.P. (2010). Larval food quality affects adult (but not larval) immune gene expression independent of effects on general condition. Mol. Ecol..

[83] Muhammad A., He J., Yu T., Sun C., Shi D., Jiang Y., Xianyu Y., Shao Y. (2022). Dietary exposure of copper and zinc oxides nanoparticles affect the fitness, enzyme activity, and microbial community of the model insect, silkworm Bombyx mori. Sci. Total Environ..

[84] Muhammad A., Zhou X., He J., Zhang N., Shen X., Sun C., Yan B., Shao Y. (2021). Toxic effects of acute exposure to polystyrene microplastics and nanoplastics on the model insect, silkworm Bombyx mori. Environ. Pollut..

[85] Koch H., Schmid-Hempel P. (2011). Socially transmitted gut microbiota protect bumble bees against an intestinal parasite. Proc. Natl. Acad. Sci. USA.

[86] Sun Z., Lu Y., Zhang H., Kumar D., Liu B., Gong Y., Zhu M., Zhu L., Liang Z., Kuang S. (2016). Effects of BmCPV infection on silkworm Bombyx mori intestinal bacteria. PLoS ONE.

[87] Chen B., Zhang N., Xie S., Zhang X., He J., Muhammad A., Sun C., Lu X., Shao Y. (2020). Gut bacteria of the silkworm Bombyx mori facilitate host resistance against the toxic effects of organophosphate insecticides. Environ. Int..

[88] Mikonranta L., Mappes J., Kaukoniitty M., Freitak D. (2014). Insect immunity: Oral exposure to a bacterial pathogen elicits free radical response and protects from a recurring infection. Front. Zool..

[89] Douglas A.E. (2017). The B vitamin nutrition of insects: The contributions of diet, microbiome and horizontally acquired genes. Curr. Opin. Insect Sci..

[90] Li T., Zhang Q., Zhang X., Wan Q., Wang S., Zhang R., Zhang Z. (2021). Transcriptome and microbiome analyses of the mechanisms underlying antibiotic-mediated inhibition of larval development of the saprophagous insect Musca domestica (Diptera: Muscidae). Ecotoxicol. Environ. Saf..

[91] Hosokawa T., Kikuchi Y., Shimada M., Fukatsu T. (2007). Obligate symbiont involved in pest status of host insect. Proc. R. Soc. B Biol. Sci..

[92] Marcinkevicius E.V., Shirasu-Hiza M.M. (2015). Message in a biota: Gut microbes signal to the circadian clock. Cell Host Microbe.

