# Supplementary material for: Effect of Diet on the Midgut Microbial Composition and Host Immunity of the Fall Armyworm, Spodoptera frugiperda
Source: Biology (Basel). 2022 Nov 1;11(11):1602. doi: 10.3390/biology11111602 (PMC9687563; doi:10.3390/biology11111602)
Supplement: Supplementary file 1 [file biology-11-01602-s001.zip › biology-1854176-supplementary.pdf]

Supplementary Figures and Tables

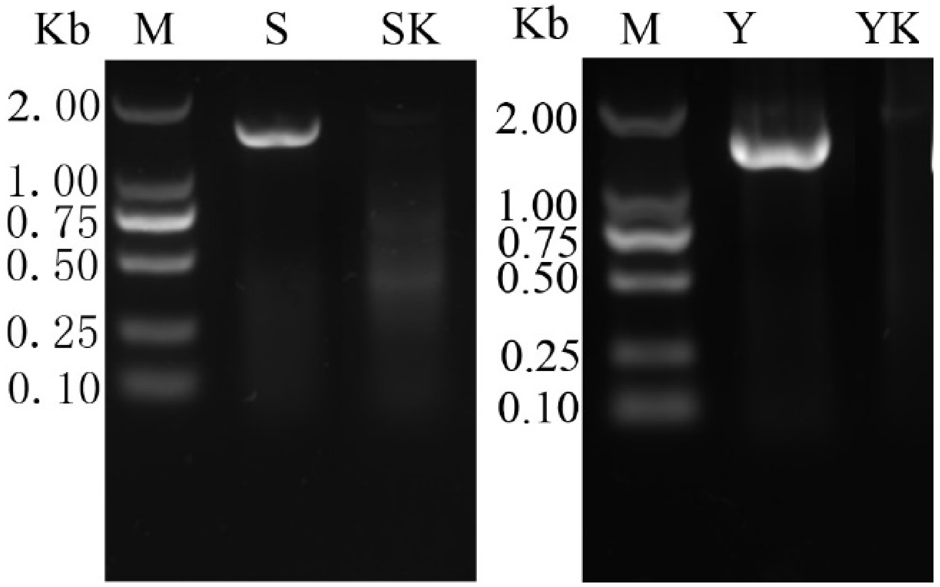

Figure S1. PCR amplification of the 16S rDNA gene. YK: field diet with antibiotics, Y: field diet without antibiotics; SK: lab diet with antibiotics, S: lab diet without antibiotics

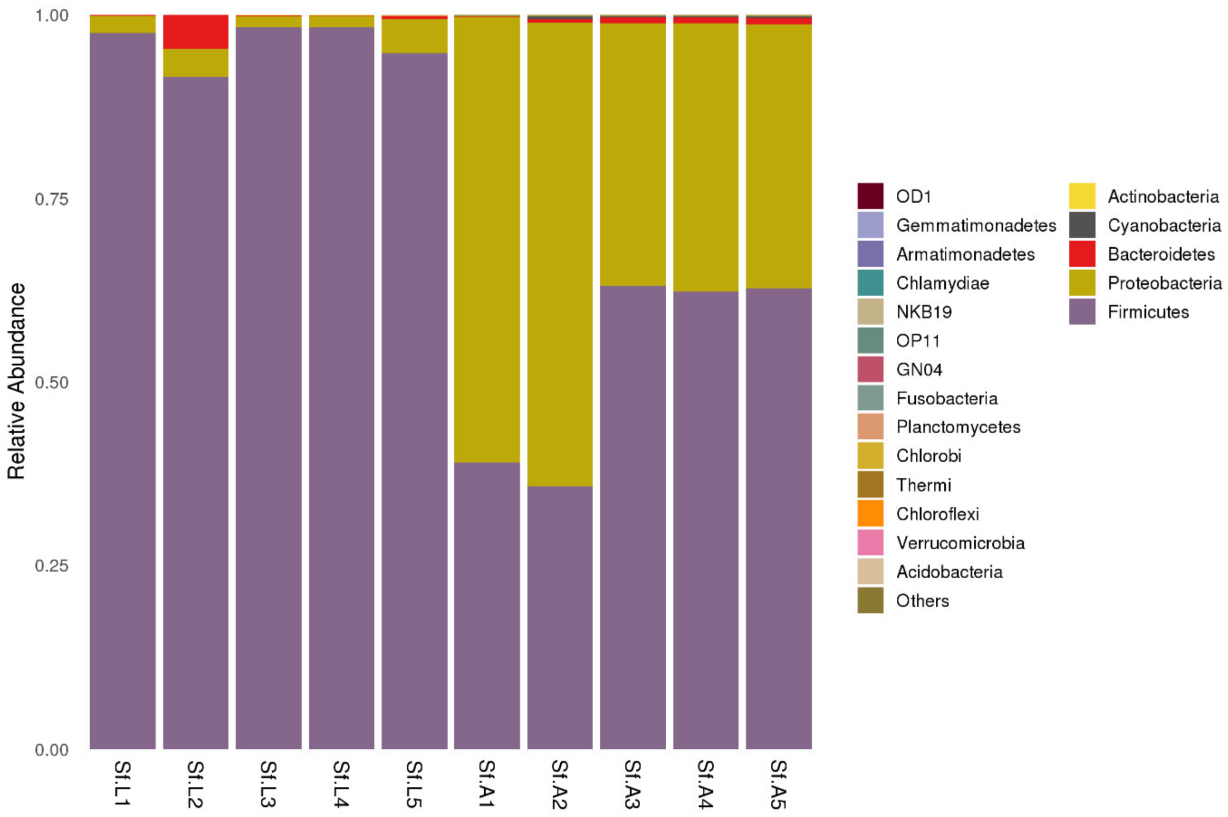

Figure S2. Relative abundance of the bacterial community at the phylum level. *S. frugiperda population feeding on corn leaf (Sf.L1,2,3,4,5); S. frugiperda population feeding on starch-rich artificial diet (Sf.A1, 2,3,4,5).*

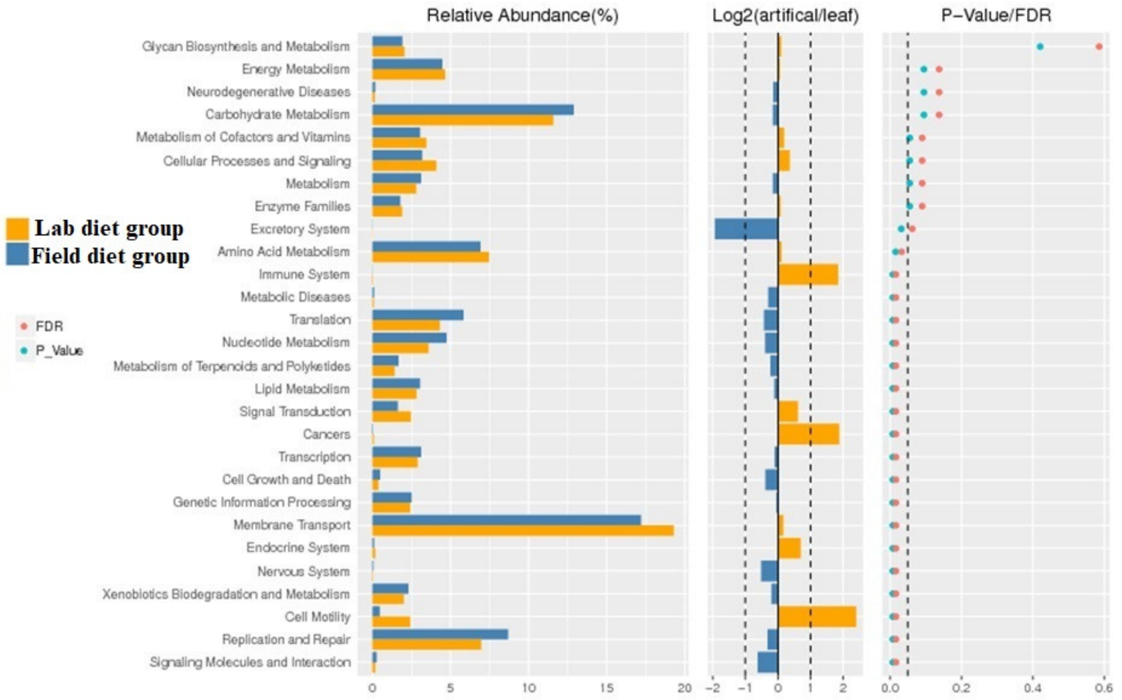

Figure S3. Inferred functions of bacterial communities associated with *S. frugiperda*. All of the predicted KEGG metabolic pathways are shown at the second hierarchical level and grouped by major functional categories. Field diet group: *S. frugiperda* population feeding on field diet (corn leaf); Lab diet group: *S. frugiperda* population feeding on lab diet (starch-rich artificial diet).

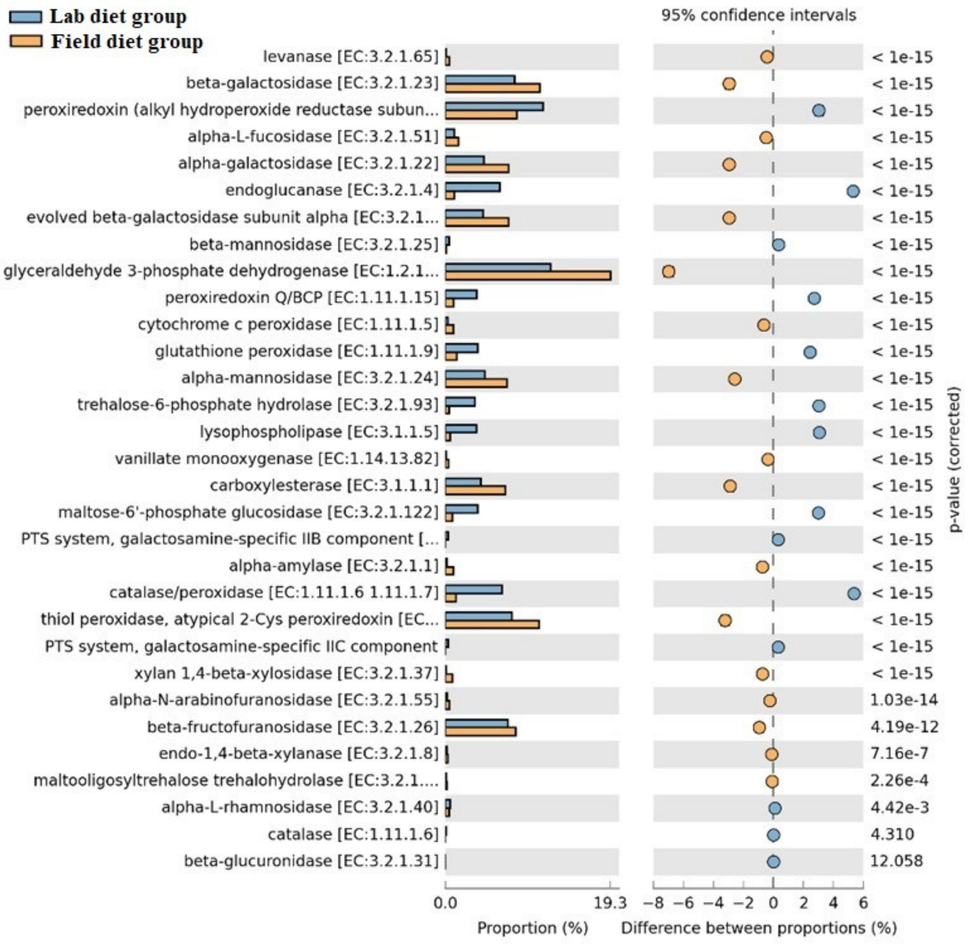

Figure S4. Inferred functions of bacterial communities associated with the plant biomass-degrading gene. Field diet group: *S. frugiperda* population feeding on field diet (corn leaf); Lab diet group: *S. frugiperda* population feeding on lab diet (starch-rich artificial diet).

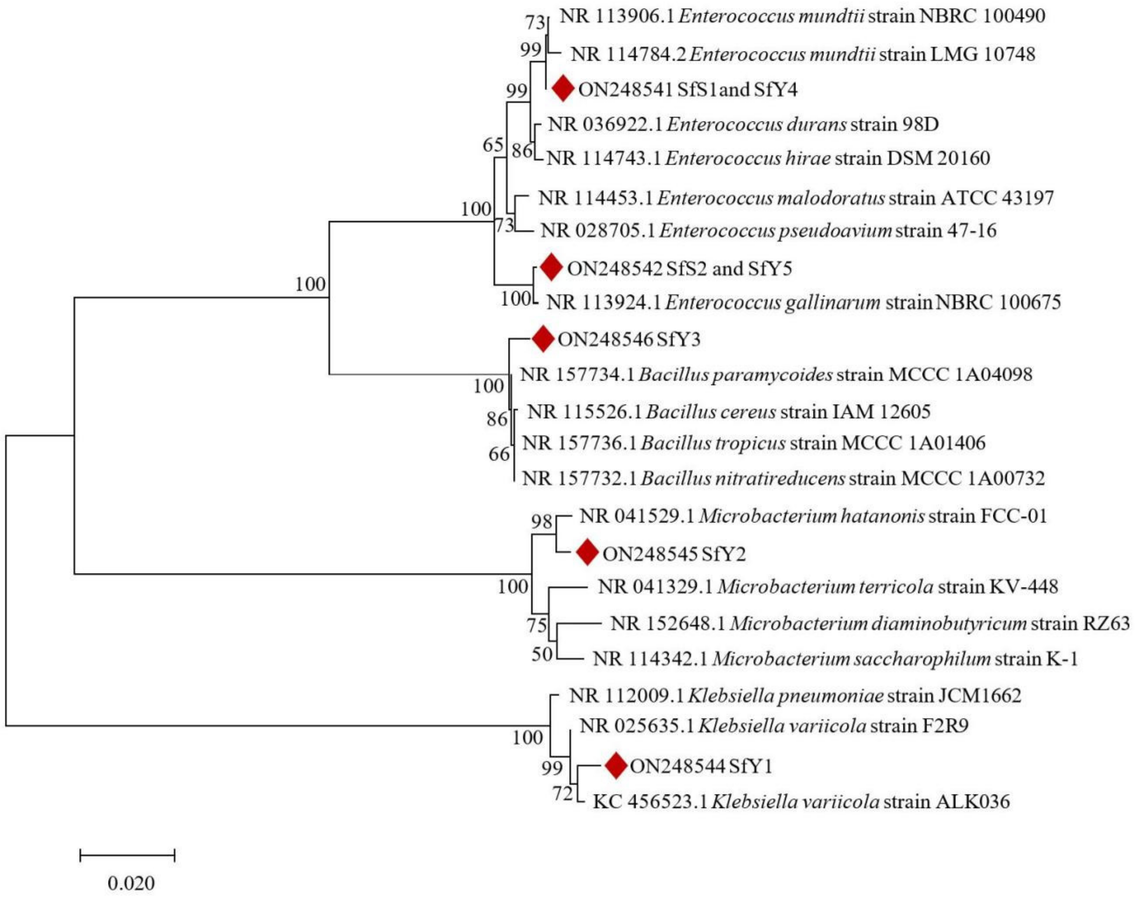

Figure S5. Phylogenetic analysis of seven isolates with related bacteria strains based on 16S rDNA sequence. Phylogeny was estimated by using the neighbor-joining method conducted in MEGA7 software. The scale 0.020 is the genetic distance. Bootstrap values (in percent) are based on 1,000 replications. Sequence GenBank accession numbers are shown in close to clade position.

Table S1. List of Primers and their sequences used in RT-qPCR.

Classification	Gene name	Primer sequence(5'------3')	
Reference	RpL18	RpL18-F	GCTCTGCACGTTACCGAGA
		RpL18-R	GCAGCAGAACGGTCTTGG
P450s	CYP9A60	CYP9A60-F	TGTACCAGCTTCTTCGGGAG
		CYP9A60-R	CGACCAGCCTGTTGTTGAAA
ABCs	ABCC2	ABCC2-F	CGATGAGGAAGGTGAGGAGAA
		ABCC2-R	CCAACACGAGGAATGCCATTA
	ABC C13-like	ABC C13-F	ACTACCTCTTGATGCTCCTACA
		ABC C13-R	AAGTTCGGCGTCCATATCTG
	ABC subG1-like	ABC subG1-F	GCCTCTGTAAGTTCGTCGTCTA
		ABC subG1-R	CGTCAACATCCTCTTCAGCAAT
GSTs	GST D7-like X1	GST D7-F	TCAACACACGATTCCGACCAT
		GST D7-R	GTAAGCATTGGCGAGGTAAGC
	GST 2-like	GST 2-F	ATCCGTATGCTGCTGGCTTA
		GST 2-R	CGTACTCCTTACCGTCAATCAC
UGTs	UGT 1-8-like	UGT 1-8-F	GAGGAGGTTCATCAATGGACTG
		UGT 1-8-R	CGTTGTGGTAAGCGTCTTCA
	UGT2B19-like	UGT 2B19-F	CGTCTTCCAAGCGGTATTCC
		UGT 2B19-R	CGTTGCCAATGATGTAGTCACT
AMPs	Gloverin-like	Gloverin-F	TCGTCTTGAAGGTCAGGCATA
		Gloverin-R	GGCGTTGTTATTGTTCCAGTTC
	Attacin-A-like	Attacin-A-F	CGGACTGACGGTGATGAAGGAA
		Attacin-A-R	CGTTGTGGTTGTCGTTGTGGAA
	Attacin-like	Attacin-F	GTTCAGTGCCATTGGAGGAG
		Attacin-R	TGTGTTGGTTAGCGAGAGTC
Other immune related genes	Cactus	Cactus-F	ACTGACACACAGACCCCACA
		Cactus-R	TCATCACAGCGAGGTGTAGC
	LITAF	LITAF-F	AACAGCAACACCAGCAACAG
		LITAF-R	AAACAAAGCCCAAGAGCAAA
	Relish	Relish-F	TATGGCACCAACAAAACGAA
		Relish-R	CGATACCACCGAACCTGACT
	16SrRNA	27F	AGAGTTTGATCCTGGCTCAG
		1492R	TACGGTTACCTTGTTACGACTT

Table S2. The summary of 16S rRNA blast results of cultivable bacteria isolates.

Strain name	GenBank Accession Number	Blast strains with the highest consistency in blast results (GenBank accession number）%
SfS1	ON248541	*Enterococcus mundtii* strain NBRC 100490（NR_113906.1）100
SfS2	ON248542	*Enterococcus gallinarum* strain NBRC 100675（NR_113924.1）99.86
SfY1	ON248544	*Klebsiella variicola* strain F2R9（NR_025635.1）99.93
SfY2	ON248545	*Microbacterium hatanonis* strain FCC-01（NR_041529.1）98.61
SfY3	ON248546	*Bacillus paramycoides* strain MCCC 1A04098（NR_157734.1）98.65
SfY4	ON248541	*Enterococcus mundtii* strain NBRC 100490（NR_113906.1）100
SfY5	ON248542	*Enterococcus gallinarum* strain NBRC 100675（NR_113924.1）99.86
